# Prolonged Beneficial Effect of Brief Erythropoietin Peptide JM4 Therapy on Chronic Relapsing EAE

**DOI:** 10.1007/s13311-020-00923-5

**Published:** 2020-09-21

**Authors:** Deeya Gaindh, Yun-Beom Choi, Michelle Marchese, Peter Dowling, Stuart Cook, Benjamin Blumberg, James H. Park, Wei Lu

**Affiliations:** 1 Neurology Service, VA Medical Center of East Orange, East Orange, NJ USA; 2 Department of Neurology, Rutgers New Jersey Medical School, Newark, NJ USA

**Keywords:** Erythropoietin (Epo), JM4 peptide, Multiple sclerosis, Experimental autoimmune encephalomyelitis (EAE), Bioluminescence imaging (BLI)

## Abstract

Potent beneficial immunomodulatory and anti-inflammatory effects of whole-molecule erythropoietin have been demonstrated in a variety of animal disease models including experimental autoimmune encephalomyelitis (EAE); however, excessive hematopoiesis limits its use in clinical applications. Our group previously generated an Epo-derived small peptide JM4 that is side-effect free and has strong neuroprotective activity without hematologic effects. Here, we investigated the long-term clinical effects of brief treatment with JM4 in chronic relapsing EAE using bioluminescence imaging (BLI) in transgenic mice containing the luciferase gene driven by the murine GFAP promoter. EAE mice treated with JM4 exhibited marked improvement in clinical scores and showed fewer disease flareups than control animals. JM4 therapy concomitantly led to markedly decreased GFAP bioluminescence in the brain and spinal cord in both acute and chronic relapsing EAE mouse models. We found a marker for toxic A1 astrocytes, complement component C3, that is upregulated in the brain and cord of EAE mice and sharply reduced in JM4-treated animals. In addition, an abnormally leaky neurovascular unit permeability was rapidly normalized within 5 days by JM4 therapy. The prolonged therapeutic benefit seen following brief JM4 treatment in EAE mice closely resemble that recently described in humans receiving pulsed immune reconstitution therapy with the disease-modifying compounds, alemtuzumab and cladribine. Our study suggests that JM4 therapy may have widespread clinical applicability for long-term treatment of inflammatory demyelinating diseases and that BLI is a useful noninvasive means of monitoring murine disease activity of the central nervous system.

**Electronic supplementary material:**

The online version of this article (10.1007/s13311-020-00923-5) contains supplementary material, which is available to authorized users.

## Introduction

Experimental autoimmune encephalomyelitis (EAE) is a T cell–mediated autoimmune disease affecting the central nervous system (CNS) in laboratory animals that leads to progressive weakness closely resembling the clinical manifestations of multiple sclerosis (MS) [1]. Due to the clinical and immunopathological similarities to MS, EAE is widely used as an animal model for the development of therapies for MS [2]. Two newly recognized FDA-approved MS therapies known as pulsed immune reconstitution therapies, alemtuzumab and cladribine, induce sustained clinical and radiographic beneficial effects following a brief treatment course and the favorable clinical response may last for many years [3].

Erythropoietin (Epo) is a pleiotropic cytokine involved in the proliferation, viability, and terminal differentiation of erythroid precursor cells [4, 5]. Whole-molecule Epo provides neuroprotection against ischemic toxicity, ameliorates brain injury, and improves memory in animal models by preventing beta-amyloid degradation [6–9]. Given the potential healing role of Epo in the development of new therapeutic approaches for neuroinflammatory disorders, research efforts remain underway to more clearly define its mechanism and its downstream effects [10]. The sequence of the hormone erythropoietin (*EPO*) gene was reported over 30 years ago and its dimeric receptor (EpoR) gene shortly thereafter [11–13]. Epo is largely produced and secreted by the kidney and signals to the red blood cell precursors to undergo vigorous proliferation in the bone marrow [14]. The dimeric EpoR is widely expressed throughout many nonerythropoietic tissues of the body including the CNS, heart, and kidney, as well as being extensively expressed on vascular endothelial cells [14]. The EpoR is found in neurons, microglia, and astrocytes; however, it is not expressed in the oligodendrocyte [15]. In addition to its critical role in red cell survival and proliferation, the Epo ligand plays an important role in tissue protection and immune regulation in many nonerythropoietic tissues. Some of the early data claimed that the Epo tissue protective effects might be mediated through activation of a heterodimer composed of EpoR combined with the β common receptor (βCR) [16]. However, Lodish et al. tested for Epo protective effects in two neuronal cell lines that contained only EpoR but were devoid of the βCR [17]. Extensive neuronal tissue protection was still fully induced, indicating that the βCR was not critical. More recently, Shing and coworkers used a variety of sensitive biophysical techniques to also show the extracellular domains of EpoR and βCR failed to directly or functionally interact [18]. Like Lodish, our group previously used the neuronal PC12 cell line to assess tissue protection using both the whole EPO molecule and the JM4 peptide in an Aβ40 cytotoxin assay [19]. Both whole-molecule erythropoietin and JM4 were fully protective in this neuronal cell line assay indicating that βCR receptor is not required for protection from Aβ40 toxicity.

Results of our preclinical studies in a short-term myelin oligodendrocyte glycoprotein (MOG) EAE mouse model revealed potent immunomodulatory effects of Epo demonstrated by marked clinical improvement in weakness, reduced mononuclear cell infiltration, and downregulation of glial major histocompatibility complex (MHC) class II expression within the inflamed CNS [20]. Although whole-molecule Epo shows therapeutic promise in the EAE model, toxicities including elevation in red cell mass, cardiovascular complications, stroke, and hypertension limit interest in its use in human clinical practice [21–23]. To avoid the side effects induced by whole-molecule Epo, our group generated a small side-effect free Epo-derived peptide, JM4, found to have robust Epo-like tissue protective properties in symptomatic EAE mice, without inducing excessive erythropoiesis [19, 24, 25].

We found that treatment of EAE mice with both whole-molecule Epo and JM4 reduced elevated mononuclear cell counts to normal, decreased dendritic cells by tenfold, and decreased proinflammatory cytokines including IL-2, IL-6, TNF-alpha, and INF-gamma [19]. Additionally, JM4 peptide was found to expand Treg cells and reduce T helper Th17-positive cells in SJL/J EAE mice [19]. Given these findings, we believe the mechanism of JM4 activity in neuroprotection closely resembles that of whole-molecule Epo through profound effects on both innate and acquired immunity, involving microglia, astrocytes, and T cells.

Bioluminescence imaging (BLI) is a sensitive quantitative imaging modality that may be utilized in EAE to noninvasively monitor neuroinflammation, predict disease onset, and follow disease flareups [26]. BLI has shown widespread clinical applicability to track the progression of beta-amyloid accumulation in Alzheimer’s mouse models and to measure prion infectivity [27, 28]. Prior work has used BLI to serially quantify glial fibrillary acidic protein (GFAP) expression, a known marker for astrocyte activity, to follow CNS neuroinflammation in transgenic mouse models, thereby serving as a paradigm for monitoring the inflammatory disease process [26, 29, 30].

Astrocyte activation is a practical biomarker that reflects the process for EAE induction and activation is observed prior to the onset of clinical symptoms [31]. Elevated GFAP expression is seen before the onset of clinical symptoms in relapsing–remitting EAE mice, and astrocyte reactivity similarly occurs in the beginning stages of MS lesion formation and persists chronically [31, 32]. In recent years, two different types of reactive astrocytes termed A1 and A2 have been identified [33, 34]. A1 astrocytes overexpress GFAP, present with upregulated expression of complement component C3, and are upregulated in MS and neurodegenerative disorders [33], in which they lead to death of oligodendrocytes and neurons. A2 astrocytes in contrast are thought to be neuroprotective [33].

In this study, we show that JM4 treatment substantially reduces long-term inflammatory effects in the acute MOG monophasic disease and in chronic relapsing–remitting proteolipid protein (PLP) induced EAE mice, and that BLI is a good biomarker for tracking the clinical course of EAE. We also demonstrate increased C3 expression in the CNS of EAE mice and show marked reduction in C3 expression in JM4-treated animals. These sustained and long-term clinical benefits in response to brief treatment with JM4 resemble those of the newly recognized immune reconstitution multiple sclerosis drugs, alemtuzumab, and cladribine.

## Methods

### Animals

Male FVB/N-Tg(GFAP-luc+/−) 53Xenmice (Xenogen Corp, Alameda, CA) (Taconic model 10501) were crossed with either female SJL/J (JAX Stock No. 000686) or albino B6(Cg)-Tyr^c-2J^/J (JAX stock no: 000058) mice purchased from The Jackson Laboratory (Bar Harbor, ME). The F1 offspring were genotyped by PCR following the manufacturer’s protocol. Only female mice between 8 and 10 weeks of age positive for GFAP-luc were used. Animal handling and care was performed in accordance with the Animal Component of Research Protocol (ACORP) guidelines at the VA New Jersey Health Care System in East Orange, NJ.

### Peptides

The myelin-derived antigen proteolipid protein peptide PLP139-151 (HSLGKWLGHPDKF) or MOG35-55 (MEVGWYRSPFSRVVHLYRNGK) was used for the induction of relapsing–remitting chronic and the monophasic forms of EAE, respectively. A nonhematopoietic (Epo)-derived short peptide fragment, JM4 28-46 (GCAEHCSLNENITVPDTKV), was used for therapy. All peptides were purchased from United Biochemical Research, Inc., WA.

### Induction of EAE and Clinical Assessment of Animals

Active EAE was induced according to a standard protocol [35]. Mice were immunized on day 0 by subcutaneous injection on both sides of the tail base with 100 μL of an emulsion composed of either PLP139-151 or MOG35-55 peptide in an equal volume of Freund’s adjuvant, supplemented with 4 mg/mL killed M. tuberculosis H37Ra (Difco Laboratories, Detroit, MI). The initial dose of antigen was 100 μg of PLP peptide for inducing relapsing EAE in SJL/J mice, or 200 μg of MOG peptide per GFAP-luc/B6(Cg)-Tyr^c-2J^/J mouse for induction of acute monophasic EAE. GFAP-luc/SJL/J mice received a second PLP antigen immunization on day 7. Immediately following these immunizations, all mice received intravenous (IV) injections of 200 ng Bordetella pertussis toxin diluted in 200-μL phosphate buffered saline (PBS) (List Biological Laboratories, Campbell, CA). The GFAP-luc/B6(Cg)-Tyr^c-2J^/J mice received an additional IV injection of 200 ng Bordetella pertussis toxin on postinoculation day 2. Animals were weighed and assessed daily for clinical signs of EAE by two blinded independent observers during the acute phase of illness and followed three times a week during the later chronic phase. The relapsing PLP GFAP-luc/SJL/J and EAE MOG GFAP-luc/B6(Cg)-Tyr^c-2J^ models were monitored for 28–30 days and were followed for nearly 6 months after immunization. The clinical scoring system used to quantify behavioral neurological deficits in the EAE mouse models (Table 1) was previously described [36].

**Table 1:** Clinical scoring system for quantifying neurological deficit in mouse EAE models

Score	Characteristics
0	Normal
1	Tail limb drop
2	Mild hind-leg weakness
3	Severe hind-leg weakness (paralysis)
4	Hind-leg paralysis plus mild front-limb involvement
5	Quadriplegic
6	Moribund or dead

### Bioluminescence Imaging

Bioluminescent signals were quantified using the *In Vivo* Imaging System 100 (IVIS; Xenogen, Alameda, CA) with a charged coupled device (CCD) camera. Three animals were imaged simultaneously. Mice received an IV injection of 1 mg/kg d-luciferin (Xenogen) 2–3 min prior to imaging and were immediately anesthetized with vaporized isoflurane for imaging. The imaging signal was quantified in units of photons per second per centimeter squared per steradian (photons/s·cm^2^/sr) using Living Image Version 3.1 (Xenogen) software and integrated over 2 min. For signal quantification, photons were obtained from a “region of interest” (ROI) that covered 2.18 cm^2^ of contiguous forebrain and 5.27 cm^2^ of spinal cord region. Bioluminescence was expressed as a ratio of the total photons value from the central nervous system ROI to the photon value obtained from an equal sized area over the left ear, used as an endogenous control. In the acute EAE model, measurements were typically taken every 1–2 days and the average of 2 consecutive days readings were used. Bioluminescence imaging was performed every other day after disease onset and twice weekly during the later chronic phase of the illness.

### JM4 Treatment

The JM4 peptide was first dissolved in distilled water to 2 mg/ml and stored at − 80 °C. Immediately before use, the peptide solution was further diluted to 5 μg/200 μl with PBS. Treatment was initiated when the bioluminescence signal of either the brain or spinal cord became significantly higher than background (usually 8–9-day postimmunization and prior to onset of clinical signs). The EAE mice were randomly divided into a JM4-treated and a sham-treated group. The JM4-treated EAE animals (4–10 per group) were injected daily with IV JM4 250 μg/kg of the peptide in PBS for 10–12 days. Control EAE animals were sham-treated with IV saline for the same time period. To evaluate the treatment effects, mice underwent BLI examination as well as clinical and histopathologic evaluation.

### Real-Time PCR

To verify the correlation between GFAP message levels and luciferase expression, total RNA from EAE mouse brain was extracted at different time points after immunization. A two-step real-time PCR was performed on an ABI 7700 Sequence Detection System (PE Applied Biosystems, Foster City, CA, USA) using the SYBR-Green I Master Kit (Roche Diagnostics, Indianapolis, IN, USA). cDNA was synthesized via RT PCR using SuperScript VILO (Invitrogen) on 2 μg of total RNA extracted with TRIzol (Invitrogen). Two microliters of 20 times-diluted RT PCR reaction solution was tested in the real-time PCR reaction. The primer pair used to amplify the GFAP transcript: forward 5′-ATGGTGATGCGGTTTTCTCTTC-3′ and reverse 5′-CACGAACGAGTCCCTAGAGC-3′, and for the luciferase transcript forward: 5′-GCTTTTGGCGAAGAATGAAA-3′ and reverse 5′-CATTCCGCATACTGAGATTT-3′. The real-time PCR was run for 40 cycles at 94 °C for 25 s, 60 °C for 25 s, and 72 °C for 45 s. Quantification was performed using the relative standard curve method described in User Bulletin #2 for the ABI Prism 7700 from PE Applied Biosystems. The product of HPRT1 (hypoxanthine-guanine phosphoribosyltransferase 1) transcript was used as an endogenous control. Standard curves were generated using 6 serial dilutions and a correlation score of > 0.99 was observed for each run. Samples were run in triplicate and the average Ct value was used for analysis. The melting temperature was studied with a dissociation curve and PCR products were verified by electrophoresis in a 1% agarose gel.

### Histopathology

JM4 peptide–treated and sham-treated EAE groups were anesthetized and perfused with ice-cold PBS, followed by 4% paraformaldehyde into the left ventricle. Spinal cords and brain were removed. Tissues were embedded in paraffin, sectioned, and stained with Luxol fast blue/periodic acid-Schiff to determine the extent of demyelination. Six-micrometer paraffin sections from the high cervical, thoracic, and low lumbosacral region of the spinal cord were placed on the same slide. Each slide contained a control normal spinal cord section, a sham-treated EAE cord, and spinal cord sections from JM4-treated EAE animals. Histopathologic examination was performed in blinded fashion. To assess acute axonal injury, SMI-32 anti-neurofilament H immunohistochemical staining was completed as described previously [27]. In brief, 6-μm sections were de-paraffinized, treated with antigen-retrieving solution, and blocked with normal horse serum to minimize nonspecific binding. The sections were first incubated with mouse SMI-32 antibody (Sternberger Monoclonals, Lutherville, MD) diluted 1:5000 in PBS overnight at 4 °C. After two 5-min PBS washes, the sections were incubated with biotinylated horse anti-mouse IgG (Vector Labs, Burlingame, CA) and then reacted with horseradish peroxidase (HRP)-avidin complex (Vector Labs) for 30 min each. Immunostaining was visualized by reacting the sections with stable diaminobenzidine tetrahydrochloride (Invitrogen) for 1 to 3 min. Excess DAB was removed by washing with distilled water and the slides were evaluated using light microscopy.

To assess the effect JM4 treatment on blood–brain barrier (BBB) integrity, spinal cord sections from acutely symptomatic MOG EAE mice were stained with IgG antibody in order to detect serum leakage. Eight MOG EAE mice were immunized as previously described and randomly divided into JM4-treated and a sham-treated group. Four JM4-treated EAE mice were treated daily with IV JM4 (5 μg/day × 6) starting on day 11 and four additional EAE animals were sham treated with IV saline. The animals were sacrificed on day 16 postimmunization by cardiac perfusion using cold PBS followed by 4% PFA. To detect IgG leakage within the EAE spinal cord, Cy3 conjugated Donkey anti-mouse IgG (H, L) antibody was used. In brief, 8-μm sections were cut, fixed in cold acetone, dried, and washed in PBS for 5 min. The sections were then incubated with IgG antibody at 1:120 (Jackson Lab, Bar Harbor, ME) for 60 min, washed with dH_2_0 5 times for 3 min, mounted with glycerol, and analyzed by fluorescent light microscopy.

Spinal cord (cut into 6–7 equal pieces from cervical to sacral) was frozen and serially cut at 8-μm-thick cross sections and collected on slides, then postfixed in acetone at − 20 °C for 10 min. Sections were first treated with Target Unmasking Fluid (Pan Path) at 90 °C for 10 min and blocked in 2% normal horse serum for 10 min. Sections were incubated overnight at 4 °C with either monoclonal rat anti-mouse C3 complement (Abcam, AB11862) at 1:50 or polyclonal rabbit anti-mouse GFAP (Dako Z03344) at 1:500. The next day, sections were washed in PBS three times for 10 min each and incubated with Cy3 or Cy2 conjugated goat anti-mouse/rat/rabbit IgG (Jackson ImmunoResearch Laboratories) at 1:120 working dilution for 1 h. Sections were washed in dH2O three times for 10 min each and imaged by fluorescence microscopy.

Immunohistochemistry images were obtained using an Olympus BX41 fluorescent microscope (Center Valley, PA, USA) fitted with a Jenoptik ProgRes MF cool CCD camera using 4× or 10× objectives. Half of each section from different spinal levels was quantified by measuring the percent area in the structure in which label exceeded a constant threshold value using Image Pro 4.0 image analysis software (BD Biosciences, Franklin Lakes, NJ).

### Statistical Analysis

Data is presented as the mean ± SEM. Two-tailed *t* tests were used to compare JM4 effects on BLI and clinical scores. The long-term effects of JM4 were studied in the relapsing EAE model by using each subject’s average clinical score from day 60 onward. Bioluminescence was measured by the number of days in which luminescence intensity in the spinal cord exceeded a threshold of 0.5 relative to the left ear. Survival data was examined using a two-tailed log-rank test. Values of *p* < 0.05 were considered significant.

## Results

### Effect of JM4 Treatment on Bioluminescence in Relapsing–Remitting and Monophasic EAE Models

#### Sustained Effect of JM4 Treatment on the PLP-Induced Relapsing–Remitting EAE Model

**Fig. 1:**
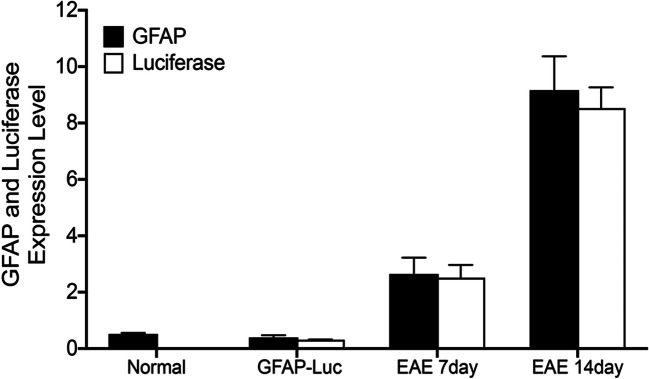
Relative expression of GFAP mRNA and luciferase in GFAP-Luc/SJL EAE mice. mRNA was extracted from SJL/J EAE mice brains at days 0, 7, and 14 postimmunization and quantified using RT PCR. A strong correlation was noted between GFAP mRNA and luciferase. SJL/J EAE mice showed increased expression of GFAP and luciferase on days 7 and 14 (*n* = 3, 6–7-fold increase at day 7; 23–25-fold increase on day 14)

Quantitative real-time PCR was used to verify the correlation between GFAP expression and luciferase expression in the GFAP-Luc/SJL EAE mouse model. RNA was extracted from SJL/J EAE mice brains at 0-, 7-, and 14-day postimmunization and two-step real-time PCR was performed. Increased expression of GFAP and luciferase was seen at days 7 and 14 (Fig. 1). A strong correlation (> 98%) was noted between GFAP and luciferase transcription (*R*^2^ = 0.9958). Following verification of the correlation between GFAP and luciferase expression, we investigated the long-term JM4 treatment effects on chronic relapsing–remitting PLP antigen-induced SJL/J EAE mice. We predicted that monitoring GFAP-luc expression with bioluminescent imaging might be a more sensitive and comprehensive indicator for determining CNS neuroinflammatory status than clinical examination. To test this prediction, we created a new light-producing FVB/N-Tg(GFAP-luc)53Xe/SJL EAE model by crossing FVB/N-Tg(GFAP-luc)53Xe with SJL/J.

**Fig. 2:**
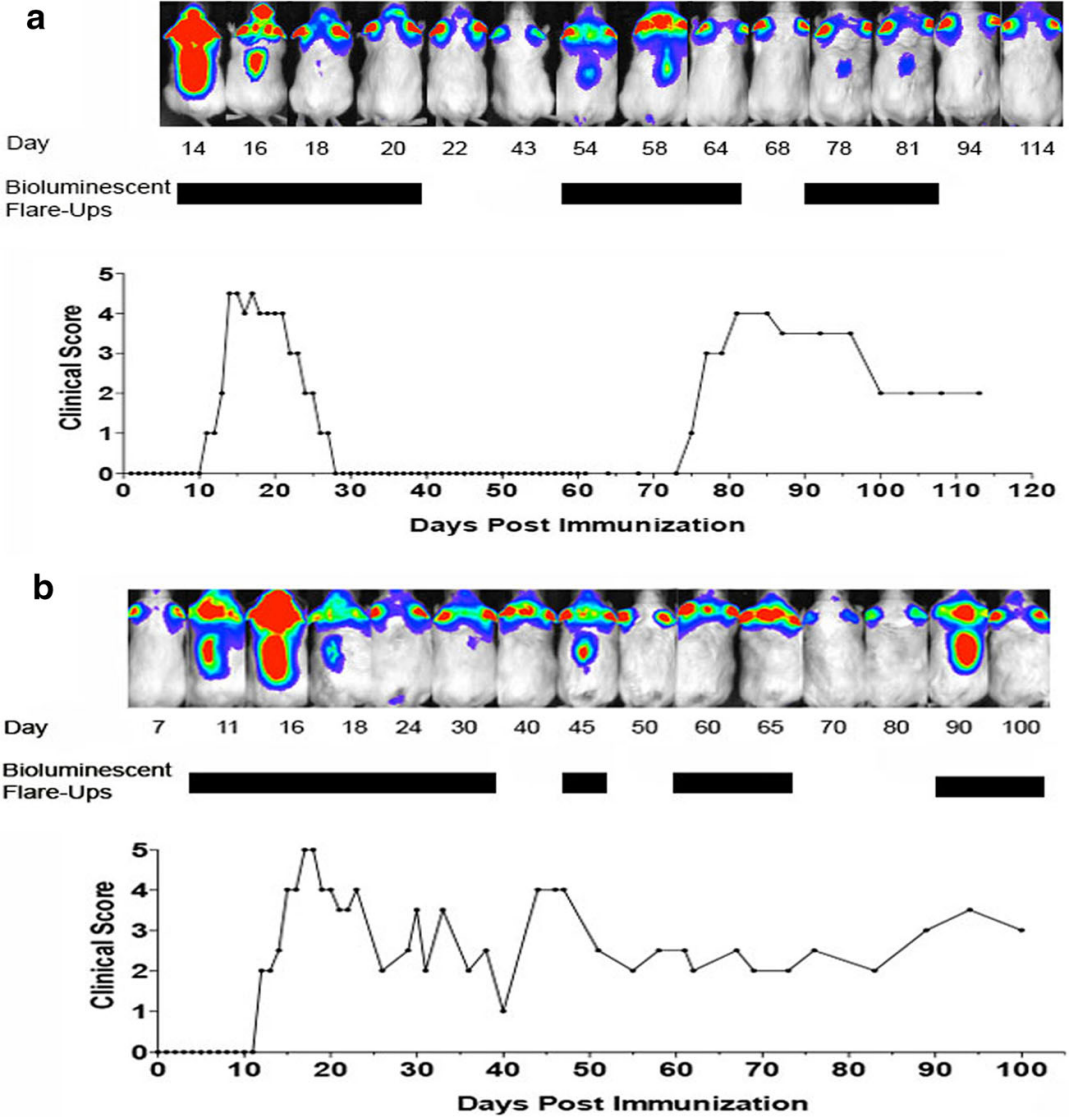
Longitudinal assessment of BLI and clinical scores in two untreated GFAP-luc/SJL relapsing–remitting EAE mice. Black bars represent bioluminescent flare ups. (**a**) Following initial clinical presentation (days 14–22), the mouse remained asymptomatic from day 28 to 73 (upper panel). Despite strong BLI enhancement, there was no clinical correlate at days 54–64. In contrast, the third episode of BLI enhancement at days 78–81 corresponded to a strong clinical relapse with severe paralysis. (**b**) In the second animal (lower panel), a correlation was consistently seen between BLI and clinical scoring. Increased BLI signal in the brain and spinal cord was associated with increased clinical deficit in three distinct episodes (days 11–30, day 45–60, days 90–100)

Female F1 offspring were immunized with 100 μg of PLP139-151 and a booster immunization was administered on day 7. The mice were evaluated by clinical exam and BLI imaging for up to 6 months. Eighty percent of PLP immunized mice developed EAE and all showed at least one relapse as assessed by clinical scoring during the 5–6-month experimental course. The first clinical signs appeared 11 ± 1.5-day postimmunization, with clinical scores reaching a peak at 14 ± 2.4 days and on average the acute peak lasted 7.5 ± 3.8 days. Bioluminescent signal above background level was detected at 9 days in 80% of animals. Increased signals were first seen in the forebrain and subsequently extended to spinal cord in 1–3 days with spinal cord signals typically peaking 1–2 days prior to the onset of clinical manifestations. The intensity of photon readings in the spinal cord correlated strongly with the clinical score. In 20% of PLP immunized mice, no clinical symptoms or increased bioluminescent signal in the brain was seen; these mice were removed from the study. Prior to a definite clinical recurrence, the animals always exhibited a substantial increase in bioluminescent signal in the spinal cord that subsequently receded to background levels over the course of a few days. Bioluminescent intensity increases in the rostral forebrain were not always followed by clinical evidence of disease (Fig. 2).

**Fig. 3:**
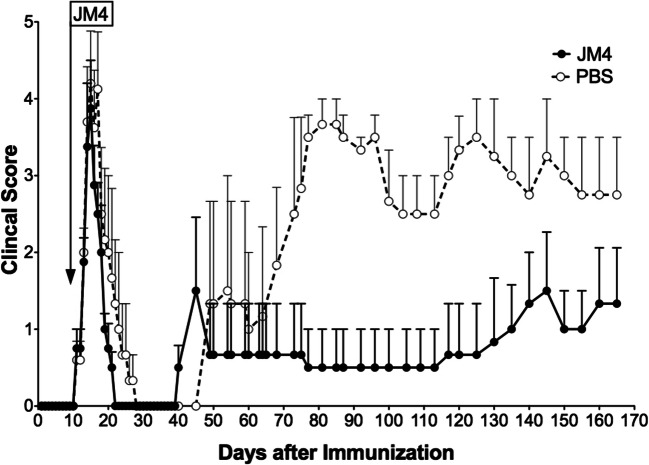
Effect of JM4 on clinical scores in long-term PLP-induced relapsing–remitting EAE. GFAP-luc/SJL mice were treated for 12 days with JM4 (5 μg IV) starting on day 9. Average clinical scores in the JM4-treated group, from day 60 onward, remained remarkably reduced compared to the sham-treated group for over 5 months (*n* = 4 JM4 treated, *n* = 5 sham treated, *p* < 0.05)

**Fig. 4:**
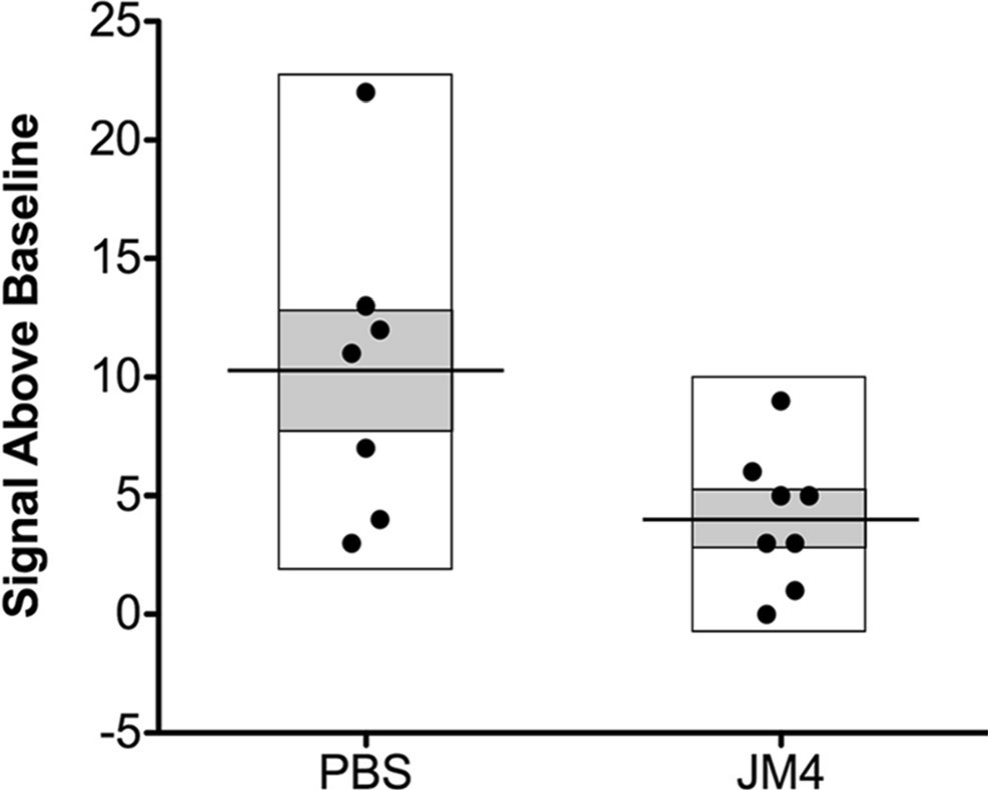
Positive treatment effect on flare ups of spinal cord bioluminescence in GFAP-luc/SJL relapsing–remitting EAE mice treated with JM4 (5 μg IV daily for 12 days). Episodes of spinal cord bioluminescent signal over background during disease course were calculated. JM4 treatment significantly decreased the number of imaging flare ups compared to flare ups in the sham-treated group (*n* = 8 JM4 treated, *n* = 7 sham treated, *p* < 0.05). Gray bars represent standard error (mean ± SEM sham treated 10.29 ± 2.447, JM4 treated 4.0 ± 1.018)

After characterizing the new light-producing SJL/J EAE mouse model on day 9, we divided the mice (4–6 animals in each experiment) into two groups, one treated with a 12-day course of IV JM4 peptide (5 μg/day) and the other sham-treated (0.9% saline IV). Clinical deficits were scored daily, and images were taken 2–3 times weekly during the acute stage and subsequently followed by imaging every 7–10 days for up to 5 months. Long-term clinical scores of JM4-treated relapsing–remitting mice remained significantly lower than sham-treated EAE mice for over 5 months (Fig. 3). Clinical deficit in JM4-treated animals rapidly improved 7 days earlier than sham-treated animals during the acute phase of the disease. The average clinical score for JM4-treated mice remained under 1 for 130 days, whereas sham-treated group scores centered around 2 for the same period. In JM4-treated relapsing–remitting EAE mice, both GFAP-luc expression and total number of disease flare-ups in either the brain or spinal cord were significantly decreased compared to sham-treated animals (Fig. 4). Treatment with JM4 led to 100% survival in relapsing–remitting SJL/J mice at 100 days, whereas only 50% of sham-treated animals survived, as analyzed by Kaplan-Meier plot (not shown).

#### Effect of JM4 Treatment on MOG-Induced Monophasic EAE Model

**Fig. 5:**
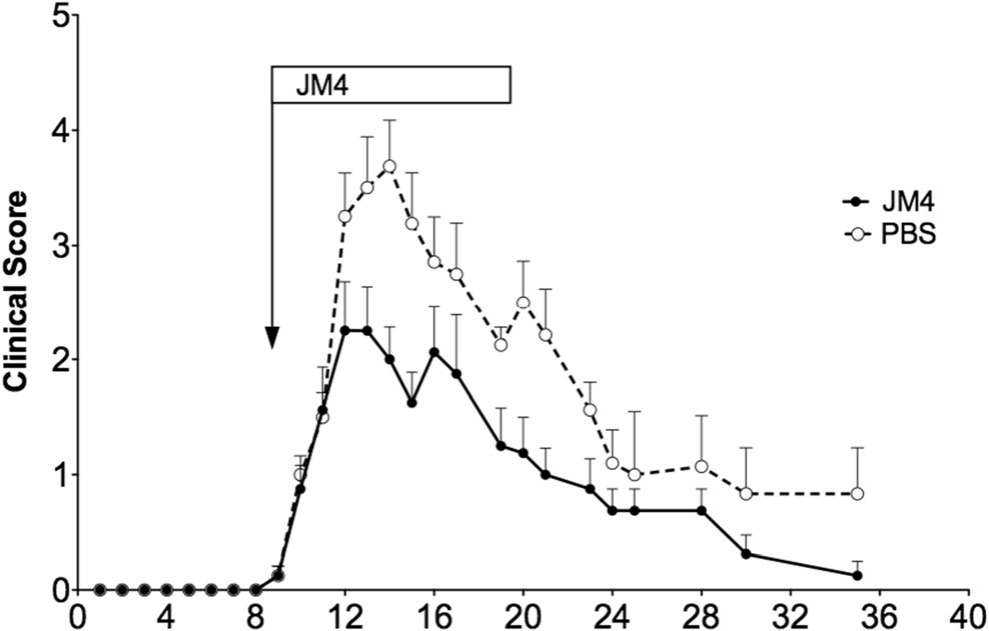
Effect of JM4 on clinical scores in JM4-treated monophasic MOG EAE mice. MOG immunized GFAP-Luc/C57 mice developed significant neurologic impairment on 11-day postimmunization. Treatment with JM4 (5 μg IV daily for 12 days) was initiated on day 9. Peak clinical scores in JM4-treated MOG animals were significantly lower compared to sham-treated MOG EAE mice (*n* = 8 JM4 treated, *n* = 8 sham treated, *p* < 0.05)

Luo et al. [17] used GFAP-luc/B6(Cg)-Tyr^c-2J^/J albino mice to study MOG-induced acute EAE and found a close correlation between GFAP bioluminescence and clinical scores. We used the GFAP-luc/B6(Cg)-Tyr^c-2J^/J model to investigate the therapeutic effects of JM4 on MOG-induced monophasic EAE. Female F1 offspring of FVB/N-Tg(GFAP-luc)53Xen crossed with B6(Cg)-Tyr^c-2J^/J were immunized with 200 μg MOG. These mice later received either JM4 250 μg/kg IV or sham treatment with 0.9% saline IV for 12 days starting on day 9 postimmunization. Disease onset and severity were assessed by clinical neurologic scoring as well as by GFAP-luc BLI. We observed a monophasic clinical course as described by Luo et al. [17] in the sham-treated EAE group. Mice first developed clinical signs on day 10 ± 0.7 and reached a maximum mean clinical score of 3.7 ± 0.4 on day 14, with approximate disease duration of 2 weeks. In contrast, EAE animals treated with JM4 showed a significant reduction in clinical score from day 12 throughout the remainder of the disease course (Fig. 5), and the maximum mean clinical score was reduced to 2.25 ± 0.3 (*p* < 0.05).

**Fig. 6:**
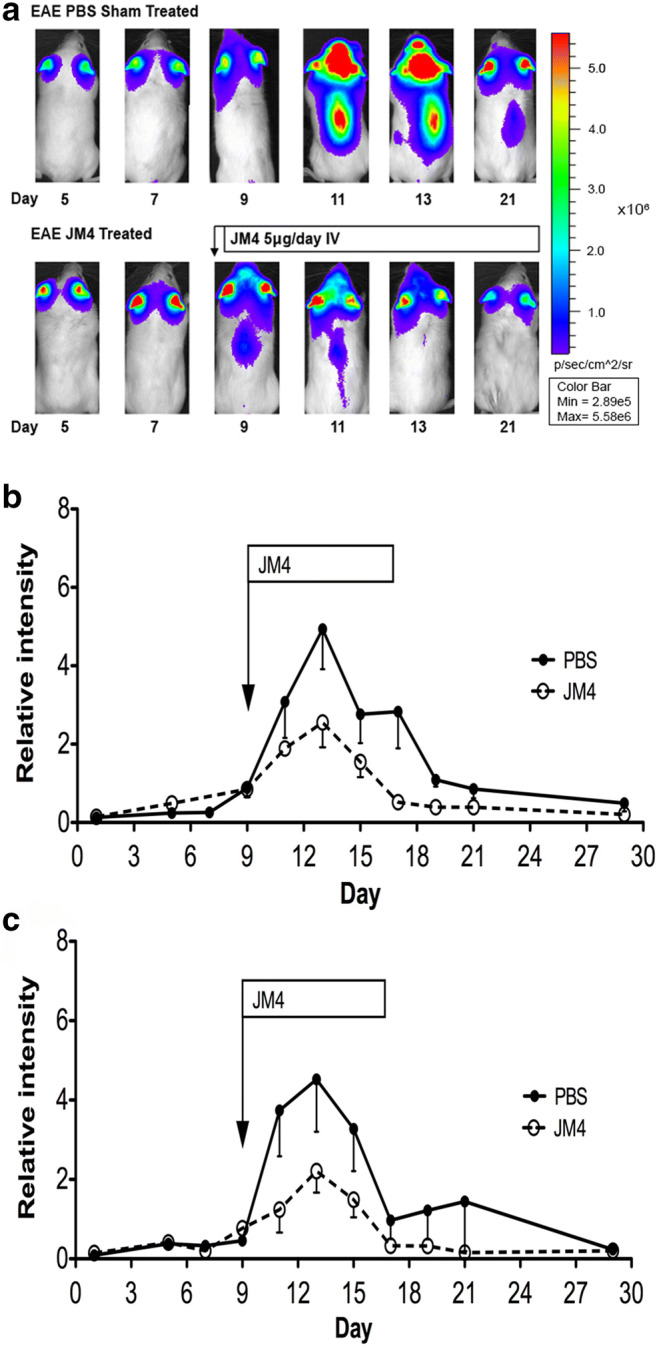
(**a**) *In vivo* imaging following treatment with JM4 in MOG-induced EAE. Sham-treated GFAP-Luc/C57 mice (upper panel) and JM4-treated mice (lower panel) were monitored using BLI over 21 days. Treatment with JM4 (5 μg IV daily for 12 days) was started on day 9. This JM4-treated animal exhibited lower spinal cord peak intensity within 2 days after treatment and virtual absence of spinal cord signal by day 4 (**b**, **c**). Relative intensity of bioluminescence in forebrain (**b**) and spinal cord (**c**) in GFAP-Luc/C57 MOG-induced EAE mice. Peak bioluminescent values were significantly lower in JM4-treated GFAP-Luc/C57 EAE mice in both forebrain and spinal cord (brain *n* = 8, *p* < 0.05; spinal cord *n* = 8, *p* < 0.05). GFAP-Luc/C57 MOG-induced EAE mice were treated with JM4 (5 μg IV daily for 12 days) starting on day 9. Relative intensity was measured as the ratio of photon intensity in the lesion compared to the left ear value

A marked positive treatment effect with JM4 was also seen by GFAP-luc BLI assessment that correlated with clinical scores (Fig. 6). In the MOG-induced EAE model, earliest detectable bioluminescent signal (ROI/Ear ratio ≥ 0.4) was noted over the forebrain as early as 7-day postimmunization. A significant signal was detected in 90% of animals by 9 days, and maximum values were observed between days 11 and 16, typically preceding onset of clinical deficit by 2–3 days. Animals with similar bioluminescent scores at day 9 were paired into JM4-treated or sham-treated EAE groups. Peak bioluminescent readings within the brain and spinal cord regions over the disease course in the JM4-treated MOG EAE group remained significantly lower than those of the sham-treated group (Fig. 6). A 3–4-fold reduction from peak disease scores (days 11–17) at days 11 to 12 was seen in JM4 treated compared with sham-treated animals. In addition, GFAP-luc signal returned to baseline 3–4 days earlier in JM4-treated animals compared to the sham-treated group.

### A1 Astrocyte Activation Represented by C3 Expression in MOG-Induced Monophasic EAE Mice Was Attenuated by JM4 Treatment

**Fig. 7:**
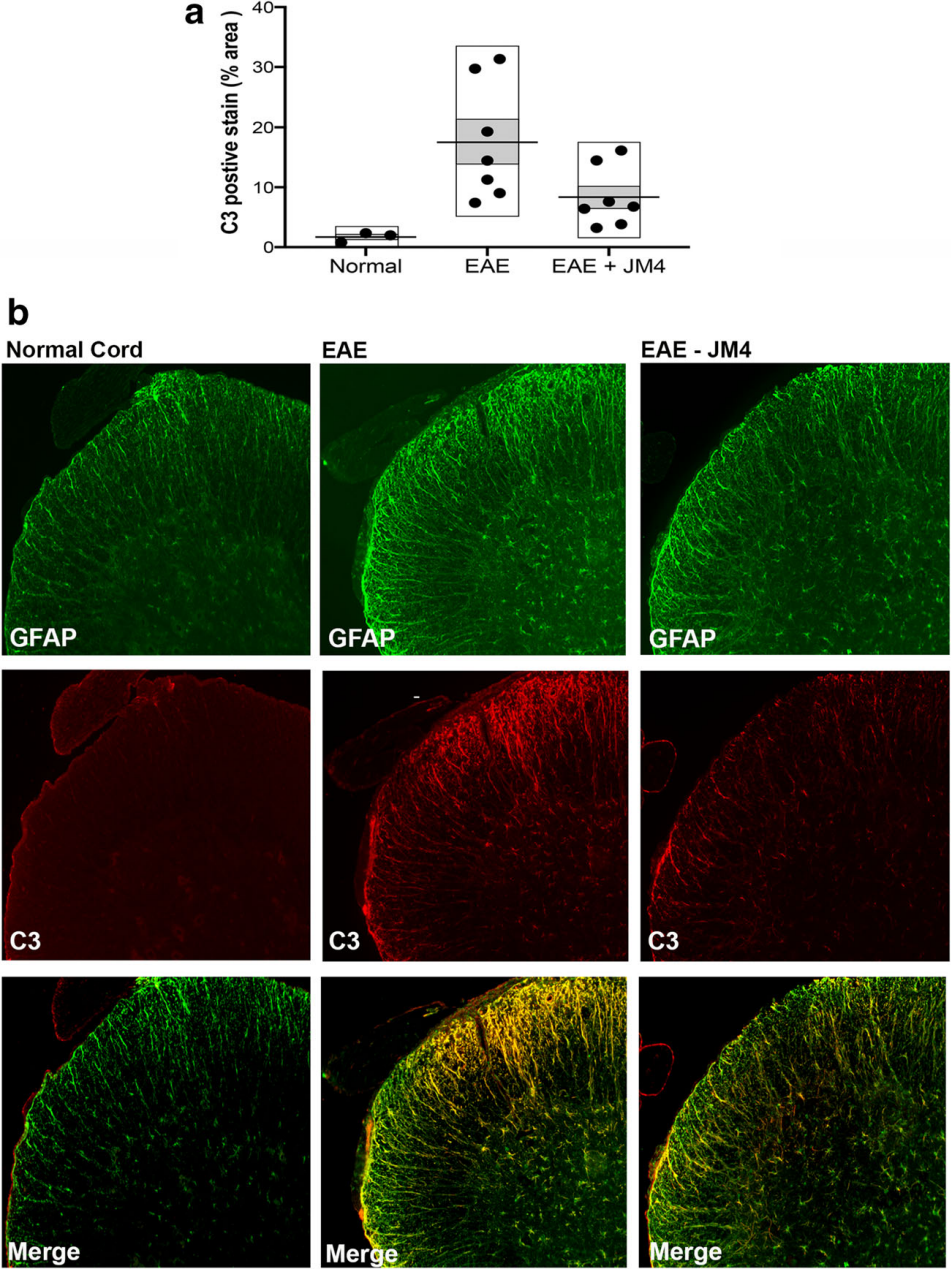
Treatment with JM4 leads to significantly reduced A1 astrocyte activation in the spinal cord of EAE mice. (**a**) Quantitative analysis showing significant upregulation of GFAP+/C3+ astrocytes in EAE mice compared to normal mice, and significant decrease in GFAP+/C3+ astrocytes immunoreactivity following treatment with JM4 in EAE mice (data represent mean ± s.e.m). (**b**) Immunofluorescence images showing reduction in both GFAP (green) and C3 (red) immunoreactivity in the spinal cord of JM4-treated EAE mice. Merged images showed most C3 immunoreactivity is found on GFAP positive astrocytes (yellow) (*n* = 7 per group, EAE 6.1 ± 0.8 versus JM4 treated 3.4 ± 1.2, *p* < 0.05, ANOVA followed by Bonferroni comparison between EAE and JM4-treated EAE mice)

MOG-induced EAE mice received either JM4 250 μg/kg IV or sham treatment with 0.9% saline IV for 12 days starting on day 9 postimmunization. To show aggressive A1 astrocyte activation in the CNS of EAE mice, we performed immunohistochemistry for complement component C3, a marker for A1 astrocytes. C3 expression colocalizing with GFAP was upregulated in the spinal cord of the MOG EAE mice 24 days postimmunization compared to sham-treated control mice. Importantly, treatment with JM4 led to a significant reduction in C3 expression (Fig. 7). These results show that JM4 treatment attenuated toxic A1 astrocyte activation by over 50% in EAE mice.

### JM4 Treatment Leads to Repair of the Blood–Brain Barrier

**Fig. 8:**
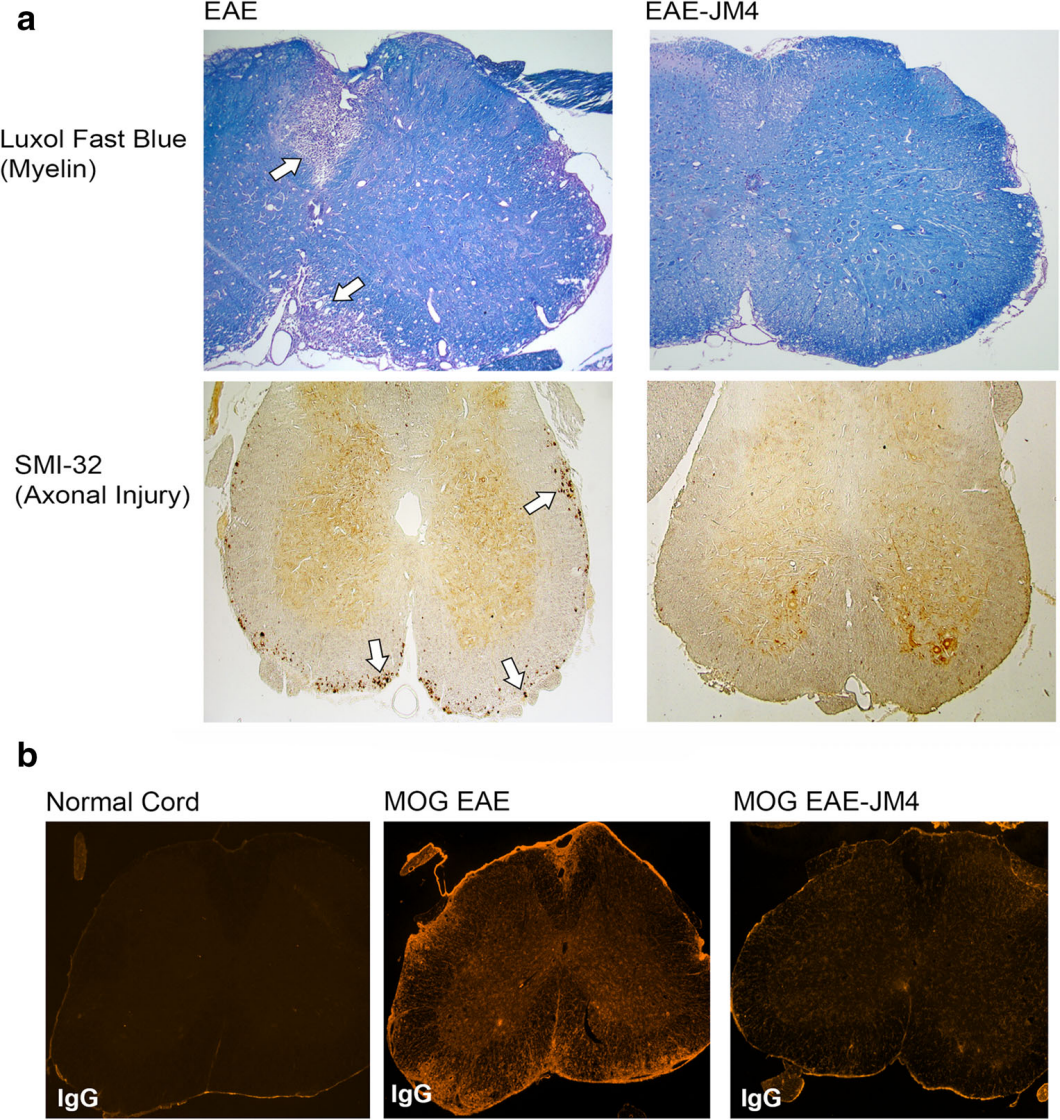
(**a**) JM4 is protective against spinal cord demyelination and axonal injury in SJL/J relapsing–remitting EAE mice. Luxol fast blue stain for myelin (top panel) in sham-treated EAE mice shows pronounced demyelination and vacuolization in the ventral white matter of the spinal cord compared to JM4-treated animals. SMI-32-stained axons (lower panel) in JM4-treated mice show a large reduction in number of injured axons rimming the periphery of the ventral spinal cord (white matter) compared to sham-treated EAE control mice. (**b**) JM4 therapy attenuates blood–brain barrier breakdown. Spinal cord sections were stained for mouse IgG to determine if serum leakage occurred. In a normal mouse spinal cord section, no IgG is detected. In contrast, saline treated EAE mice showed increased immunoreactivity most notably seen in the white matter of the spinal cord. Treatment with JM4 profoundly reduced the amount of IgG immunoreactivity in EAE cord back to close to normal

Sham-treated EAE mice showed pronounced demyelination in the white matter of the spinal cord, whereas the number of injured axons lining the periphery of the ventral spinal cord in JM4-treated mice was reduced (Fig. 8). To assess the effect of JM4 treatment on BBB integrity, spinal cord sections from acutely symptomatic MOG EAE mice were stained with IgG antibody to detect serum leakage. In the normal mouse spinal cord, no IgG was detected, whereas saline-treated acutely symptomatic EAE mice showed substantially increased immunoreactivity most notably in the white matter of the spinal cord. A short course of treatment with JM4 led to marked restoration of the barrier function with virtually no IgG detectable by immunofluorescent staining of JM4-treated acute EAE spinal cord (Fig. 8).

## Discussion

Our hybrid BLI/clinical study demonstrates the profound immunomodulatory benefit of our small Epo-derived peptide, JM4, in both relapsing–remitting and monophasic mouse models. In both models, the positive clinical and imaging effects of JM4 treatment were sustained without the negative hematogenic side effects associated with whole-molecule Epo.

Traditional treatments for relapsing–remitting multiple sclerosis, including oral and injectable disease modifying therapies, have usually demonstrated beneficial effects that are limited to the duration of therapy. An emerging class of treatments for MS called immune reconstitution therapies shows markedly prolonged clinical benefits that last well beyond the brief treatment course. Two immune reconstitution compounds have been recognized: alemtuzumab and cladribine; these compounds deplete lymphocytes, class switched and unswitched memory B cells, and lead to a reduction in relapse rate and gadolinium enhancing lesions as seen on brain magnetic resonance imaging (MRI) in MS patients. With both medications, the prolonged clinical benefit of therapy is often retained for months to years after the time of active treatment [3]. Our findings that short-term therapy with JM4 leads to sustained clinical benefits and downregulation of astrocyte activation as monitored by BLI for at least 5 months, point to JM4 as a promising therapy for achieving long-lasting benefits akin to the immune reconstitution therapies.

In the experiments shown here, relative GFAP-luc expression was noted to increase over time in the EAE transgenic mouse model, reinforcing the previously recognized concept of GFAP, the classic astrocyte marker, as a useful biomarker of neuroinflammation [26]. These outcomes were similar to previously studied acute EAE models showing increased GFAP mRNA levels coinciding with clinical symptoms and inflammation [35–37]. In the relapsing–remitting SJL/J EAE mouse model, enhanced bioluminescent abnormalities were observed earlier and more frequently than clinical deficits, enabling us to noninvasively monitor the effect of treatment at multiple time points with greater accuracy prior to the appearance of clinical symptoms. These findings suggest that BLI is often more sensitive than clinical assessment in mice. Similarly, prior work using BLI reported early detection of EAE lesions 5–7 days before the development of clinical neurologic impairment [26].

Analogous to the use of BLI for monitoring treatment response in EAE mouse models, MRI has been utilized for diagnostic purposes and to monitor disease response to therapy in MS patients. Similar to the findings of this current study, acute MS lesions seen on MRI may often precede clinical signs and symptoms [38, 39]. Interestingly, our results showed that bioluminescent intensity increases in the rostral brain were not always followed by clinical evidence of disease. These findings are reminiscent of the well-recognized clinico-radiological paradox in MS, in which radiographic lesion load seen on MRI often does not correlate with the extent of clinical disability [40, 41].

As anticipated, BLI appeared to be of greater utility in the assessment of disease status in the chronic relapsing–remitting EAE model. Although the average daily intensity of the bioluminescent signal was not significantly different between the JM4- and sham-treated groups, the number of flareups in JM4-treated animals in which GFAP expression was above background was always substantially less than that seen with sham-treated animals (Fig. 4). This data shows that a 10–12 day course of treatment with JM4 halts the onset of severe EAE in the acute phase and markedly reduces the number of inflammatory recurrences in chronic EAE animals for more than 5 months. By clinical scoring, 50% of JM4-treated SJL/J animals remained relapse-free throughout the 5-month experiment, whereas only 20% of sham-treated mice remained relapse-free. Notably, JM4 therapy for 12 days resulted in 100% survival of SJL/J EAE animals, whereas half of the sham-treated SJL/J EAE animals died.

Here, we present *in vivo* BLI and histopathological lines of evidence to support that JM4’s CNS intracellular mechanism operates by promoting the neuroprotective A2 astrocyte over the cytotoxic C3-expressing A1 astrocyte population and by rapidly repairing the leaky neurovascular unit. This sustained shift from A1 to A2 astrocytic population is reasoned to be a principal mechanism for JM4’s beneficial effect. Because microglial activation is known to induce A1 reactive astrocytosis, JM4 may suppress the development of M1 macrophages or microglia. Characterizing the identity of the cell surface receptor(s) for JM4, whether it is preferentially expressed on microglia or another cell type membrane, may shed more mechanistic insight on the intracellular signaling mechanism responsible for the A1 to A2 population shift. This notion of shifting the astrocytic population from A1 to A2 is consistent with the reported presence of toxic A1 reactive astrocytes in MS and other neurodegenerative disorders [33, 42, 43]. Likely, the sustained beneficial immunomodulatory effects of JM4 derive not only from promoting CNS astrocytic diversity towards A2 but also the inducement of a favorable peripheral anti-inflammatory cytokine profile, normalization of the disrupted blood–brain barrier, downregulation of MHC class II expression, reduction of T helper 17 cells, and expansion of T-regulatory cells in lymphoid tissue [19, 20, 25]. Further characterization of intracellular pathways responsible for shifting astrocyte types in the brain of EAE mouse models is required for the future development of effective drugs.

In this current work, we show that a short-term treatment pulse with JM4 lasting only 7–14 days led to sustained and long-term clinical benefit lasting at least 5 months in the chronic relapsing EAE mouse. Similarly, we anticipate that a short course of JM4 will lead to prolonged clinical benefits without side effects in the treatment of MS patients. If this is the case, our drug JM4 adds another promising option to the class of newly developing pulsed immune reconstitution therapies for the treatment of MS.

## Electronic Supplementary Material

ESM 1 (PDF 1225 kb)
